# Morphology control of exciton fine structure in polar and nonpolar zinc sulfide nanorods

**DOI:** 10.1038/s41598-017-09812-y

**Published:** 2017-08-24

**Authors:** Sotirios Baskoutas, Zaiping Zeng, Christos S. Garoufalis, Gabriel Bester

**Affiliations:** 1 Materials Science Department, University of Patras, 26504 Patras, Greece; 2 Department of Chemistry, University of Hamburg, D-20146 Hamburg, Germany; 3 The Hamburg Centre for Ultrafast Imaging, Luruper Chaussee 149, D-22761 Hamburg, Germany

## Abstract

Electron-hole exchange interaction in semiconductor quantum dots (QDs) splits the band-edge exciton manifold into optically active (“bright”) and passive (“dark”) states, leading to a complicated exciton fine structure. In the present work, we resolve by atomistic million-atom many-body pseudopotential calculations the exciton fine structure in colloidal polar and nonpolar zinc sulfide (ZnS) nanorods (NRs). We explore that polar NRs with high symmetry exhibit vanishing fine structure splitting (FSS), and are therefore ideal sources of entangled photon pairs. In contrast, nonpolar NRs grown along $[\mathrm{11}\bar{{\bf{2}}}0]$ and $[\mathrm{10}\bar{{\bf{1}}}1]$ directions with reduced symmetries have significant FSS, which can even reach up to a few mili electron volts. However, such large FSS can be effectively minimized to a few micro electron volts, or even less, by a simple morphology control.

## Introduction

Quantum dots (QDs) are important components for modern quantum information devices including single photon and entangled photon sources for quantum teleportation, quantum cryptography and distributed quantum computation^1–6^. This is largely motivated from the expectations that QDs are efficient light emitters that can possibly be tuned to emit at the telecom wavelength. The photons emitted from the QDs are a result of excitonic recombination, which is associated with the electron-hole exchange coupling. Compared to bulk materials, the strong quantum confinement of charge carriers in QD systems enhances the electron-hole wave function overlap, giving rise to a strong electron-hole exchange interaction. This strong electron-hole exchange interaction splits the band-edge exciton manifold into few optically active (“bright”) and optically passive (“dark”) exciton states, resulting in an intricate exciton fine structure. The energetic difference between the first dark and first bright exciton states is defined as the dark-bright splitting, which has a significant effect on the optical properties of band-edge excitons and leads to a pronounced temperature and magnetic field dependence of radiative decay^7^. The splitting in the bright neutral exciton states is commonly defined as fine structure splitting (FSS). The appearance of the FSS becomes the major obstacle in many applications exploiting QDs. As an example, FSS causes two distinguishable paths in the biexciton |XX〉–exciton |X〉–ground state |0〉 cascade recombination process, which is detrimental for the generation of polarization entangled photon-pair necessary for quantum information applications. Moreover, the presence of the FSS hints at a reduced symmetry of the zero-dimensional quantum emitters with an associated fast electron spin relaxation and a mixing of exciton spin states, undesirable for the applications of QDs in spintronics, spin-photonics and spin-based quantum information processing. However the existence of FSS that has been studied extensively in epitaxial QDs, has not been stressed in colloidal QDs. Therefore, in order to explore the potential of colloidal QDs for device applications, the understanding and the control of exciton fine structure are pivotal.

Due to their low fabrication costs and high quantum efficiency at room temperature, colloidal QDs based on wet chemistry synthesis have been demonstrated to be promising candidates for numerous applications. Among them are electrooptical devices based on the electroabsorption phenomenon^8, 9^ and photonic applications in different fields such as health, energy, environment and aerospace (ref. 10 and references therein). Contrary to epitaxial based QDs which exhibits single photon emission only at cryogenic temperatures^11, 12^, single colloidal QDs based on group II-VI compound exhibit photon antibunching at room temperature and even above^12–14^. Therefore, extensive studies have been undertaken about the exciton fine energy splitting in group II-VI QDs^7, 15–22^. Although the size dependence has been fairly well understood, knowledge about the effect of morphology on exciton fine structure is rather limited. In this contribution, we explore the exciton fine structure of colloidal wurtzite zinc sulfide (ZnS) nanorods (NRs) and show how the fine structure energy splitting can be controlled by the NRs’ morphology. Zinc sulfide (ZnS) is an important luminescent material with wide applications including light-emitting diodes^23^, electroluminescene^24^, displays^25^ and lasers^26^. The wurtzite phase of bulk ZnS is known as a high temperature phase (>1024 °C [ref. 27]). However, it exhibits a higher ionization transition rate than its zinc blende counterpart and therefore has a higher optical gain^28^. Furthermore, ZnS NRs with wurtzite phase can be fabricated at much lower temperatures^29^, even at room temperature^30, 31^, with selective orientations along either $[0001]$ polar direction or along $[11\bar{2}0]$ ^32^ and $[10\bar{1}1]$ ^33^ non-polar directions.

The NRs considered herein are characterized by diameter *D* and length *L*. They are cut from the bulk material with approximately cylindrical shape by using the experimental structure parameters (see ref. 21), leading to a realistic atomic description. The resultant point group symmetries of the polar and nonpolar NRs are shown in Table 1. The surface dangling bonds are passivated by a high-band-gap artificial material, as successfully practised previously^21, 22, 34–36^. The total number of atoms in the considered NRs ranges from a few hundred atoms to a few tens of thousands of atoms without passivation. A total of 72000 atoms including passivation has been considered for our largest NR. The single particle calculations are based on the plane wave atomistic empirical pseudopotential method with recently well-tested pseudopotentials^21, 22^, taking strain, band coupling, coupling between different parts of the Brillouin zone and spin-orbit coupling into account. The correlated excitonic states are calculated by the screened configuration interaction (CI) approach using all possible singly excited determinants constructed from 24 hole states and 4 electron states, thus taking correlation into account. The Coulomb and exchange integrals are calculated from the atomic wavefunctions and are screened by the phenomenological microscopic model proposed by Resta^37^. The optical dipole matrix elements are calculated within the dipole approximation and the oscillator strength is calculated via Fermi’s golden rule^38^.

**Table 1:** Symmetry analysis of the exciton states generated from HOMO and LUMO single-particle states of $[0001]$-, $[11\bar{2}0]$-, $[10\bar{1}1]$ ZnS nanorods.

Orientation	Point Group	Single Particle		Exciton manifold (HOMO ⊗ LUMO)
		LUMO	HOMO	
$\mathrm{[0001}]$	*C*_3*v*_	Γ_4*c*_	${{\rm{\Gamma }}}_{5v}\oplus {{\rm{\Gamma }}}_{6v}$	${{\rm{\Gamma }}}_{3}^{\ast }\oplus {{\rm{\Gamma }}}_{3}$
$\mathrm{[11}\bar{2}\mathrm{0]}$	*C*_*s*_	${{\rm{\Gamma }}}_{3c}\oplus {{\rm{\Gamma }}}_{4c}$	${{\rm{\Gamma }}}_{3v}\oplus {{\rm{\Gamma }}}_{4v}$	${{\rm{\Gamma }}}_{2}\oplus {{\rm{\Gamma }}}_{2}\oplus {{\rm{\Gamma }}}_{1}\oplus {{\rm{\Gamma }}}_{1}$
$\mathrm{[10}\bar{1}1]$	*C*_1_	${{\rm{\Gamma }}}_{1c}\oplus {{\rm{\Gamma }}}_{2c}$	${{\rm{\Gamma }}}_{1v}\oplus {{\rm{\Gamma }}}_{2v}$	${{\rm{\Gamma }}}_{1}\oplus {{\rm{\Gamma }}}_{1}\oplus {{\rm{\Gamma }}}_{1}\oplus {{\rm{\Gamma }}}_{1}$

The symmetry of HOMO, LUMO and of the resulting exciton manifold are given by the double group representations of the corresponding point group (second column), including spin-orbit coupling. Γ_3_-exciton is doubly degenerate, while Γ_1_ and Γ_2_-excitons are singly degenerate.

## Exciton fine structure in [0001] polar ZnS nanorods

**Figure 1:**
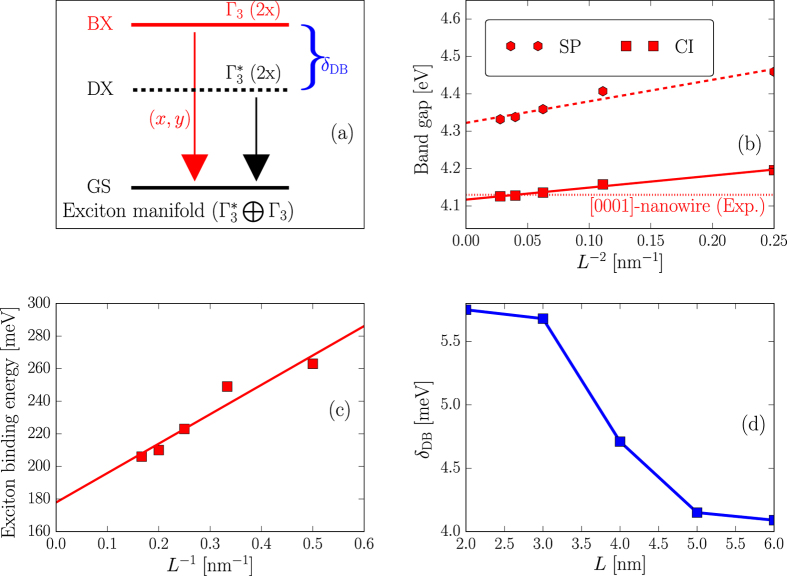
(**a**) Band-edge exciton fine structure of [0001] polar ZnS NRs. It consists a doubly degenerate (2x), in-plane polarized bright exciton (BX) state (Γ_3_-exciton) and a doubly degenerate (2x) dark exciton (DX) state (${{\rm{\Gamma }}}_{3}^{\ast }$-exciton) decaying to the ground state (GS). (**b**) Single-particle band gap (SP) and optical band gap (CI) as a function of the inverse square length (*L* ^−2^) of polar ZnS NRs with diameter *D* = 2 nm. The dotted line represents the corresponding experimental optical band gap of [0001] polar ZnS nanowire (*L* ^−2^ → 0) with diameter *D* = 2 nm [ref. 31]. (**c**) Exciton binding energy as a function of the inverse length (*L* ^−1^) of ZnS NRs with diameter *D* = 2 nm. (**d**) Dark-bright splitting as a function of the length of ZnS NRs with diameter *D* = 2 nm. The dashed and/or solid lines in (**b**–**c**) represent linear fits.

We first consider polar ZnS NRs with diameter *D* = 2 nm and *C*_3*v*_ point group symmetry. Such ultrathin single crystal NRs have been fabricated at room temperature using a catalyst-free colloidal chemistry strategy proposed in ref. 31. We find that the highest occupied molecular orbital (HOMO) state belongs to ${{\rm{\Gamma }}}_{5v}\oplus {{\rm{\Gamma }}}_{6v}$ representations of *C*_3*v*_ double group (cf. Table 1). Using a projection onto bulk technique^21, 39^, which gives us the access to the envelope functions and the Bloch function parentage of the atomic wavefunctions for each state, such a HOMO state turns out to have an *S*-like envelope function and has mainly *A*-Bloch band character with Γ_9*v*_ symmetry. The lowest unoccupied molecular orbital (LUMO) state belongs to the Γ_4*c*_ representation (cf. Table 1). It also has an *S*-like envelope function but originates from the lowest bulk conduction band with Γ_7*c*_ symmetry. The band-edge exciton manifold stems nearly purely from the HOMO–LUMO transition, consisting of a doubly degenerate dark exciton state at lower energy and a doubly degenerate, in-plane polarized bright exciton state at higher energy (cf. Fig. 1(a)). Both exciton states have Γ_3_ symmetry (cf. Table 1). This doubly degenerate Γ_3_ bright exciton state gives rise to a vanishing FSS, therefore making these high symmetry polar NRs as ideal sources of entangled photon pairs. Similar proposal has been suggested for $\mathrm{[111}]$ grown epitaxial zinc-blende QDs and heterostructure quantum wires^40, 41^, and has been experimentally realized by fabricating QDs on a high symmetry crystallographic (111) substrate^42, 43^. The double degeneracy of this bright exciton state keeps intact even with elongation of the NRs along the growth direction.

Both the single particle (SP) band gap ${E}_{g}^{{\rm{SP}}}$ and optical band gap ${E}_{g}^{{\rm{CI}}}$ are found to decrease from a nearly spherical shape (e.g., *L* = *D*) to the nanowire limit (e.g., *L* → ∞, cf. Fig. 1(b)). In agreement with a simple particle-in-a-box model, the gaps scale roughly with the inverse of the square of the NR length (e.g., *E*_*g*_ ∝ *L* ^−2^). A linear fit of the numerically obtained optical gap as a function of *L* ^−2^ delivers the nanowire limit at ${{E}_{g}^{{\rm{CI}}}|}_{L\to \infty }=4.12\,{\rm{eV}}$, which reproduces exactly the experimental data from ref. 31 (cf. dotted line in Fig. 1(b)). Subtracting the HOMO-LUMO single particle gap from the correlated gaps gives the exciton binding energy, which is plotted as a function of *L* ^−1^ in Fig. 1(c). It turns out that the binding energy ${E}_{b}^{X}\propto 1/L$. Similar scaling law has been found for the diameter dependence of ${E}_{b}^{X}$ in spherical colloidal ZnS QDs (e.g., ${E}_{b}^{X}\propto \mathrm{1/}{D}^{0.92}$ ref. 21]) and of spherical colloidal CdSe QDs (e.g., ${E}_{b}^{X}\propto \mathrm{1/}{D}^{0.76}$ [ref. 44]). Such a scaling law produces the limiting value of ${E}_{b}^{X}$ in ZnS nanowire at 179 meV (cf. solid line of Fig. 1(c)), significantly larger than the bulk value of 41 meV^45^ at room temperature, which is a consequence of 2D quantum confinement. We finally study the dark-bright splitting *δ*_DB_. The dark and bright exciton states can be easily identified experimentally by cryogenic-temperature fluorescence lifetime measurements. *δ*_DB_ can then be deduced from the temperature dependence of the recombination dynamics, when the populations between the bright and dark states become redistributed (e.g., cf. refs 7, 20 and 46). Figure 1(d) shows *δ*_DB_ as a function of the rod length *L*. It is found that *δ*_DB_ for the NRs considered herein ranges from 4.5 meV to around 6 meV, which are typical values for group II-VI QDs (~1–20 meV). The reduction in the electron-hole wave function overlap leads to a decrease of *δ*_DB_ as a function of *L*.

## Exciton fine structure in $[\mathrm{11}\bar{{\bf{2}}}0]$ and $[\mathrm{10}\bar{{\bf{1}}}1]$ non-polar ZnS nanorods

**Figure 2:**
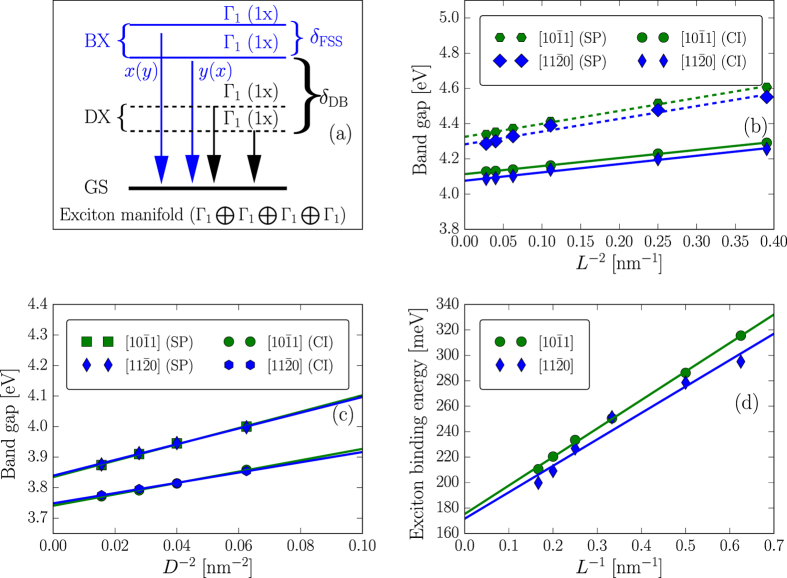
(**a**) Band-edge exciton FSS of $\mathrm{[10}\bar{1}1]$ non-polar ZnS NRs. It consists two singly degenerate (1x), in-plane polarized bright exciton (BX) states (Γ_1_-exciton) and two singly degenerate (1x) dark exciton (DX) state (Γ_1_-exciton) decaying to the ground state (GS). (**b**) Single-particle band gap (SP) and optical band gap (CI) as a function of the inverse square length (*L* ^−2^) of $\mathrm{[11}\bar{2}0]$ and $\mathrm{[10}\bar{1}1]$ non-polar ZnS NRs with diameter *D* = 2 nm. (**c**) Single-particle band gap (SP) and optical band gap (CI) as a function of the inverse square diameter (*D* ^−2^) of the non-polar ZnS NRs for length *L* = 4 nm. (**d**) Exciton binding energy as a function of the inverse length of the non-polar ZnS NRs with D = 2 nm. The dashed and/or solid lines in (**b**–**d**) represent linear fits.

Compared to polar NRs with high symmetries, NRs grown along non-polar directions exhibit reduced symmetries. As shown in Table 1, $\mathrm{[11}\bar{2}0]$-NRs have *C*_*s*_ symmetry with only two symmetry operations: the identity operation and a reflection through a mirror plane. Both the HOMO and LUMO states belong to ${{\rm{\Gamma }}}_{3}\oplus {{\rm{\Gamma }}}_{4}$ representations, and have an *S*-like envelope function with elongation along the growth direction. On the other hand, $\mathrm{[10}\bar{1}1]$ oriented NRs have no symmetry operations besides the identity (*C*_1_ point group). Both the HOMO and LUMO states belong to the ${{\rm{\Gamma }}}_{1}\oplus {{\rm{\Gamma }}}_{2}$ representations, and also have an *S*-like envelope function with elongation along the growth direction. Similar to the polar NRs, the HOMO states of both non-polar NRs have a parentage mainly from bulk *A*-band with Γ_9*v*_ symmetry and the LUMO states originates mainly from the lowest bulk conduction band with symmetry Γ_7*c*_. The band-edge exciton manifold of both non-polar NRs purely originates from the HOMO-LUMO transition, consisting of two singly degenerate dark exciton states at lower energies and two singly degenerate bright exciton states at higher energies (cf. Fig. 2(a)). For $\mathrm{[11}\bar{2}0]$-NRs, the dark states belong to the Γ_2_ representation, while the bright states belong to the Γ_1_ representation. In principle, all the states in $\mathrm{[10}\bar{1}1]$-NRs are bright by optical selection rules and belong to the Γ_1_ representation. However, the lowest two states have an oscillator strength a few orders of magnitude weaker than the upper two states and are therefore nearly dark states. The separation between the two dark states ranges from a few *μ*eV to a few tens of *μ*eV, depending on the size and on the length-to-diameter aspect ratio of the NRs.

Increasing the rod length *L* naturally leads to the decrease of the band gap, approaching the quasi 2D (nanowire) limits (e.g., *L* → ∞, cf. Fig. 1(b)). On the other hand, increasing the rod diameter *D* also causes a decrease in the band gaps, but approaching the quasi 1D (quantum well) limits (e.g., *D* → ∞, cf. Fig. 1(c)). Both the SP and optical gaps roughly scale as *E*_*g*_ ∝ 1/*L* ^2^ and *E*_*g*_ ∝ 1/*D* ^2^, respectively. These scalings are independent of the growth orientations. The gaps of $\mathrm{[11}\bar{2}0]$ and $\mathrm{[10}\bar{1}1]$ oriented non-polar NRs are found to be very comparable to their equally-sized polar counterparts (cf. Figs 1(b) and 2(b)). However, $\mathrm{[11}\bar{2}0]$ non-polar NRs exhibit a slightly smaller (~few tens of meV) gaps than the $\mathrm{[10}\bar{1}1]$ oriented ones, independent of the length-to-diameter aspect ratio. This remains true for the exciton binding energy (cf. Fig. 2(d)). The binding energy of $\mathrm{[11}\bar{2}0]$ NRs appears to be slightly smaller than that of the $\mathrm{[10}\bar{1}1]$ ones. Like the polar NRs, the binding energy of non-polar NRs also scales nearly as ${E}_{b}^{X}\propto 1/L$ (cf. Fig. 2(d)) and ${E}_{b}^{X}\propto 1/D$.

**Figure 3:**
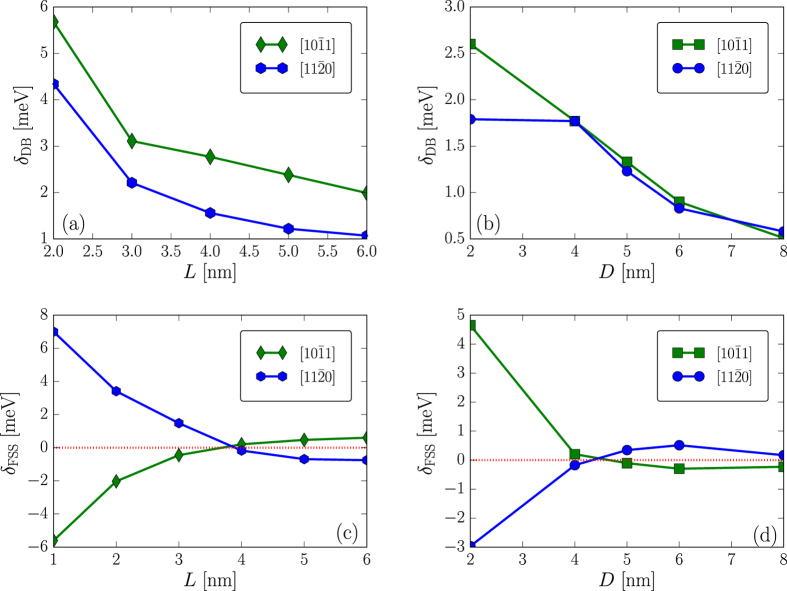
Dark-bright splitting *δ*_DB_ as a function of rod length *L* for *D* = 2 nm (**a**), and as a function of rod diameter *D* for *L* = 4 nm (**b**), of $\mathrm{[10}\bar{1}1]$ and $\mathrm{[11}\bar{2}0]$ nonpolar ZnS nanorods. Fine structure splitting as a function of rod length *L* for *D* = 4 nm (**c**), and as a function of rod diameter *D* for *L* = 4 nm (**d**), of $\mathrm{[10}\bar{1}1]$ and $\mathrm{[11}\bar{2}0]$ nonpolar ZnS nanorods. The solid lines represent linear fits. The dotted lines in (**c**,**d**) represent the vanishing fine structure splitting.

The length and diameter dependent dark-bright splitting *δ*_DB_ is shown in Fig. 3(a,b), respectively. *δ*_DB_ is found to decrease both as a function of *L* and as a function of *D*. $\mathrm{[10}\bar{1}1]$ NRs turn out to exhibit larger *δ*_DB_ than $\mathrm{[11}\bar{2}0]$ ones, at least from a nearly spherical shape to the quasi 2D (or 1D) limit. However, *δ*_DB_ in both oriented nonpolar NRs is found to be much smaller than that in the equally-sized polar one. We finally examine the FSS in these nonpolar NRs. Contrary to the high symmetry polar NRs, the bright exciton states in nonpolar NRs with reduced symmetries are non degenerate (cf. Table 1) and FSS can reach up to a few meV (cf. Fig. 3(c,d)), being extremely detrimental for the possible device applications relying on a vanishing FSS. However, *δ*_FSS_ appears be highly dependent on the length-to-diameter aspect ratio (cf. Fig. 3(c,d)). There is a critical aspect ratio at which the two in-plane polarized bright exciton states experience a level anti-crossing. The absolute value of *δ*_FSS_ therefore takes a minimum value. This is attributed to the variation of the degree of mixing between the bulk A- and B-bands in the HOMO state. This mixing is found to be minimal at the optimal aspect ratio. This admixture of bulk hole bands in the dot HOMO state is already known to have a profound effects on the FSS^47, 48^. We further find that this critical aspect ratio is dependent on the size of the NRs. It is around *ρ* = 1 for *D* = 2 nm and shifts to a slightly smaller value around *ρ* = 0.9 for a larger diameter (e.g., *D* = 4 nm). Based on our numerical results, *δ*_FSS_ can be largely reduced to few *μ*eV around the optimal aspect ratios. For example, |*δ*_FSS_| = 12.4 *μ*eV for *L* = 6 nm and *ρ* = 0.8 which is the largest system we could handle. In this case, the HOMO state has ~94% parentage from bulk A-band and only ~4% parentage from bulk B-band. *δ*_FSS_ is found to scale roughly as *δ*_FSS_ ∝ 1/*L* and *δ*_FSS_ ∝ 1/*D* ^2^, irrespectively of the growth orientations (cf. Fig. 3(c,d)).

To summarize, we have studied the FSS in polar and nonpolar ZnS nanorods. We find that the gaps and exciton binding energies appear to be very comparable in equally sized polar and nonpolar nanorods. The dark-bright splitting is found to be larger in polar nanorod than in the nonpolar cases. Polar nanorods, due to their high symmetry, exhibit a vanishing fine structure splitting, therefore being ideal sources of entangled photon pairs. Conversely, nonpolar nanorods with reduced symmetries present significant fine structure splitting which can reach up to few meV. However, it can be minimized to few *μ*eV by choosing a suitable length to diameter aspect ratio. The results presented here may be applicable for other group II–VI or even III–V wurtzite quantum dot systems. They might suggest new ways of manipulating the exciton recombination dynamics in colloidal quantum dot systems, and are useful for ongoing quantum information technologies.
